# Efficient spike communication in the MUSIC multi-simulation framework

**DOI:** 10.1186/1471-2202-12-S1-P79

**Published:** 2011-07-18

**Authors:** Ekaterina Brocke, Mikael Djurfeldt

**Affiliations:** 1 School of Computer Science and Communication, KTH, S-100 44 Stockholm, Sweden; 2 INCF, Karolinska Institutet, S-171 77 Stockholm, Sweden

## 

MUSIC is a standard API and software library allowing large-scale neuronal network simulators, or other applications, to exchange data within a parallel computer during runtime [1]. It promotes interoperability between models written for different simulators and allows models to be re-used to build a larger model system, a multi-simulation. In addition, it allows for independent development of pre- and post-processing tools, for example for scientific visualization. A prototype implementation of the API in the form of a C++ library was released under the GPL license in early 2009. The simulators NEST, Neuron and MOOSE are adapted for the MUSIC library. Work in progress includes extension of MUSIC to support multi-scale multi-simulations [2] and connecting MUSIC-enabled applications to robotic hardware [3].

In the MUSIC prototype implementation, spikes are communicated using pair-wise MPI_Send/MPI_Recv. While this scales well for one-to-one-like connectivity, there are cases where such a communication scheme might not use machine resources optimally, e.g. for all-to-all-like connectivity in a simulation running on a large number of MPI processes. Here we report on recent developments of the library providing an alternative communication algorithm based on collective MPI communication. We present benchmark results comparing the scaling of the two communication algorithms for different topologies of connectivity.

**Figure 1:**
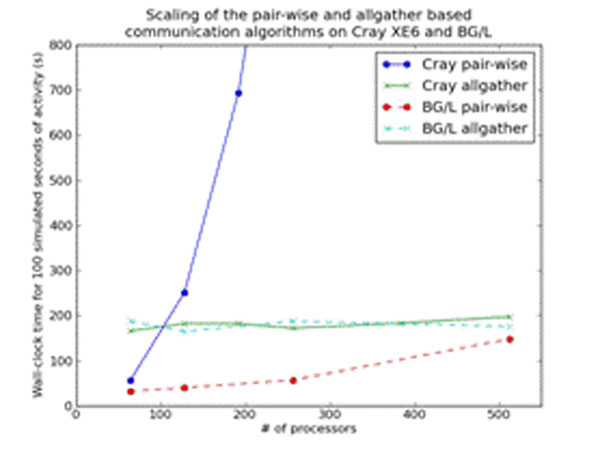
Scaling of multi-simulation with two connected populations.

In order to put maximal stress on the MUSIC implementation, we used artificial neuronal populations lacking their own dynamics (no computation) so that spike communication could be studied in isolation. We ran a multi-simulation where a simulation A communicated spikes to a simulation B. The simulations A and B consisted of 100000 neurons each. Every neuron in B received spikes from 10000 randomly selected neurons in A, each spiking at a frequency of 10Hz. Buffered spikes were communicated every 5 ms. Figure 1 shows wall-clock time for 100 simulated seconds for the two algorithms on two computer architectures. For reference, the wall-clock time for 128 processors on the BG/L (165 s) is 1.4 % of the wall-clock time for simulation of 100 s of the symmetric multi-simulation benchmark in [1] which includes computation of the neuronal dynamics. The scaling of the pair-wise algorithm on the Cray system might be possible to improve by allocating nodes within short network distance.

We describe two communication algorithms used in MUSIC and give a quantitative evaluation of their scaling performance. Preliminary results indicate that the new allgather-based algorithm scales better than the pair-wise algorithm for all-to-all connectivity. It also performs better than the pair-wise algorithm on the Cray XE6 with more than 100 processors.
